# Antigen-Engaged B Cells Undergo Chemotaxis toward the T Zone and Form Motile Conjugates with Helper T Cells

**DOI:** 10.1371/journal.pbio.0030150

**Published:** 2005-05-03

**Authors:** Takaharu Okada, Mark J Miller, Ian Parker, Matthew F Krummel, Margaret Neighbors, Suzanne B Hartley, Anne O'Garra, Michael D Cahalan, Jason G Cyster

**Affiliations:** **1**Howard Hughes Medical Institute and Department of Microbiology and Immunology, University of CaliforniaSan Francisco, CaliforniaUnited States of America; **2**Department of Physiology and Biophysics, University of CaliforniaIrvine, CaliforniaUnited States of America; **3**Department of Neurobiology and Behavior, University of CaliforniaIrvine, CaliforniaUnited States of America; **4**Department of Pathology, University of CaliforniaSan Francisco, CaliforniaUnited States of America; **5**Department of Immunobiology, DNAX Research InstitutePalo Alto, CaliforniaUnited States of America; University of MinnesotaUnited States of America

## Abstract

Interactions between B and T cells are essential for most antibody responses, but the dynamics of these interactions are poorly understood. By two-photon microscopy of intact lymph nodes, we show that upon exposure to antigen, B cells migrate with directional preference toward the B-zone–T-zone boundary in a CCR7-dependent manner, through a region that exhibits a CCR7-ligand gradient. Initially the B cells show reduced motility, but after 1 d, motility is increased to approximately 9 μm/min. Antigen-engaged B cells pair with antigen-specific helper T cells for 10 to more than 60 min, whereas non-antigen-specific interactions last less than 10 min. B cell–T cell conjugates are highly dynamic and migrate extensively, being led by B cells. B cells occasionally contact more than one T cell, whereas T cells are strictly monogamous in their interactions. These findings provide evidence of lymphocyte chemotaxis in vivo, and they begin to define the spatiotemporal cellular dynamics associated with T cell–dependent antibody responses.

## Abstract 2

Interactions between B and T cells in intact lymph nodes are monitored with two-photon laser scanning microscopy.

## Introduction

The antigen-specific interaction between B cells and helper T cells in secondary lymphoid organs is an essential step in T-dependent humoral immune responses [1]. These early B cell–T cell (B-T) interactions occur in the border area between follicles and T zones after B and T cells are antigen primed in B cell follicles and in T cell zones, respectively. During cognate B-T interaction, B cells present a specific antigen to helper T cells and receive cytokine signals requisite for their survival, proliferation, and differentiation [2]. The relocation of antigen-engaged B and T cells to the boundary from two adjacent but distinct microenvironments is believed to contribute to the prompt encounter of B and T cells with cognate-antigen specificity [1,3].

The localization of antigen-engaged B cells at the B-zone–T-zone (B/T) boundary, which was initially discovered by tracking hapten-specific memory B cells [4], has been well characterized for naive cells by examining the distribution of immunoglobulin-transgenic (Ig-tg) B cells at various times after antigen exposure [5]. These immunohistochemical studies established that the relocalization process is rapid, being complete within 6 h. A recent study showed this relocalization is driven by upregulation in B cells of CCR7 —the receptor for T-zone chemokines CCL21 and CCL19 [6]. However, these experiments have not established whether movement to the boundary occurs by directed, chemotactic movement or by random migration followed by retention by adhesive interactions at the boundary.

Antigen-specific B-T conjugates in the boundary area have been revealed by immunohistochemical analysis of Ig-tg B cells and T cell receptor (TCR)–transgenic CD4^+^ T cells in spleen and lymph node sections [7,8]. When equal numbers of antigen-specific cells were given, cognate B-T contacts were more frequent than noncognate B-T contacts in lymph node snapshots [7]. The dynamics of B-T interactions and knowledge of the spatiotemporal organization of molecules at the B-T interface have also been studied by imaging living cells on cover slips, [2,9], but it is unclear how these observations relate to events in vivo.

Recently, approaches have been developed to image living cells within intact lymphoid tissues [10–14]. Two-photon microscopy provides advantages over other imaging approaches, including a greater imaging depth in tissues and less phototoxicity; this technique has been used to image the dynamic nature of naive lymphocytes and the interactions between T cells and dendritic cells (DCs) [13–18]. Imaging has been performed both in explanted lymph nodes incubated at 37 ^o^C with oxygen-perfused medium [11,16–18] and by intravital microscopy of lymph nodes [13,14]. Naive lymphocytes, antigen-primed T cells, and DCs showed very similar motility in these two experimental configurations. These studies have revealed that T lymphocytes move in lymph node T zones at 10–12 μm/min, and B cells move within follicles at about 6 μm/min, with both cell types exhibiting what appears as a random walk. Surprisingly, despite the many studies of lymphocyte chemotaxis in vitro and our understanding of chemokine requirements for lymphocyte homing and architecture in the lymphoid organs, evidence of directional migration of lymphocytes has not yet been obtained from ex vivo or in vivo microscopy of lymph nodes [19]. Furthermore, although much has recently been discovered about the properties of T cell–DC interactions [14,16–18,20,21], no information is yet available on the dynamics of B-T interactions during T cell–dependent antibody responses.

In this study we used two-photon microscopy of explanted mouse lymph nodes to study the behavior of antigen-primed B cells and to track their interactions with helper T cells during the first stages of a T cell–dependent antibody response. We show that relocation of antigen-engaged B cells to the B/T boundary is achieved by random migration far from the follicle boundary in combination with directional migration in the region extending up to approximately 140 μm from the boundary. Directional migration was dependent on CCR7 expression by the B cells and a gradient of CCL21 was detected extending into the follicle. Furthermore, we have tracked the dynamics of B-T encounters and demonstrate that cognate B-T interactions can last for at least 1 h, whereas noncognate interactions last only a few minutes. Antigen-specific B-T conjugates are usually monogamous and migrate extensively in the interfollicular region at approximately 9 μm/min.

## Results

### Directional Migration of Antigen-Engaged B Cells to the B/T Boundary

**Figure 1:**
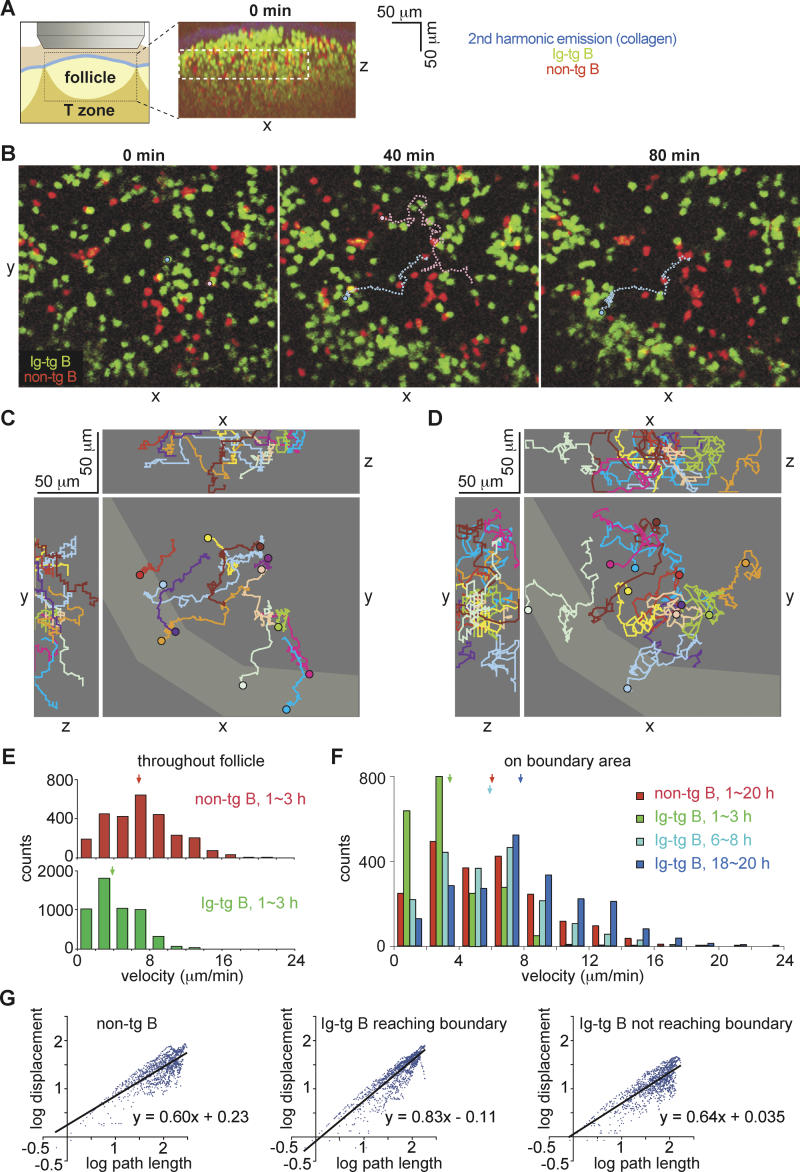
Antigen-Engaged B Cells Reduce Random Motility and Migrate Toward the Follicle–T Zone Boundary (A) On the left is a diagram showing the region of the inguinal lymph node that was imaged. The right panel shows the *xz* projection view of an image stack collected immediately prior to time-lapse imaging, demonstrating the location of a B cell follicle containing transferred B cells (green and red). The collagen-rich lymph node capsule is visualized by second harmonic emission (blue). The dashed white rectangle shows the region used in the time-lapse image analysis. (B) Time-lapse images of HEL-engaged Ig-tg B cells (green) clustering at the follicle–T zone boundary and naive non-tg B cells (red) in the follicle. The 0-min image is approximately 1 h after antigen exposure (see Video S1). The pathways of an Ig-tg B cell (light blue circle and dotted line) and a non-tg B cell (pink circle and dotted line) are traced as examples. The traced non-tg B cell moved out of the imaging stack at the 57.5-min timepoint. Scale as in (C). (C and D) Tracks of antigen-engaged Ig-tg (C) and naive non-tg (D) B cells in the *xy-, xz-,* and *yz*-planes. The boundary as defined by the area of Ig-tg B cell accumulation at 120 min is shown in lighter gray. Tracks cover 30 to 112 min (C) and 30 to 57 min (D). Circles indicate the end point of tracking. (E) Velocity distribution for naive (upper histogram) and antigen-engaged (lower histogram) B cells. Data are shown for 44 Ig-tg cells and 31 non-tg cells. Medians are indicated by arrows. (F) Velocity distributions for non-tg B cells at 1–20 h (red, *n* = 25) or Ig-tg B cells at 1–3 h (green, *n* = 29), 6–8 h (light blue, *n* = 21), or 18–20 h (dark blue, *n* = 39) after antigen injection. The data are for cells imaged in the boundary regions. Medians are indicated by arrows. (G) Ratios of log (displacement) to log (path length) of naive B cells (20 cells), antigen-engaged B cells that moved to the boundary (22 cells), or antigen-engaged B cells that did not move to the boundary (22 cells). Points indicate data for individual cells collected at cumulative 30-sec time intervals. The formula for each regression line is indicated. The correlation coefficients, *R* ^2^, were 0.63, 0.80, and 0.67, respectively. Data are from three experiments and are representative of 12 to ∼18 cells from each recording.

To investigate the migration dynamics of antigen-engaged B cells, we transferred fluorescently labeled hen egg lysozyme (HEL)-specific Ig-tg B cells (green) and nontransgenic (non-tg) B cells (red) into syngeneic mice, allowed 1 d for the cells to equilibrate within lymphoid tissues, and then tracked migration of the cells in inguinal lymph nodes excised from the recipient animals 1 h after intravenous HEL injection. By flow cytometric analysis of dissociated lymph node cells, the B cell receptors on the Ig-tg B cells were shown to be fully occupied by HEL antigen (data not shown). Extensive immunohistochemical analysis of the distribution of Ig-tg B cells in lymphoid tissues isolated at various times after HEL injection has established that the HEL-engaged B cells accumulate at the boundary of the follicle and T zone, including in interfollicular regions [5,6,22]. With our current microscope configurations, two-photon imaging through the capsule was restricted to depths of approximately 200 μm. This was too shallow to permit extensive imaging of the follicle boundary distal to the capsule, but it did allow analysis of boundary regions at the sides of follicles, which typically corresponded to interfollicular regions (Figures 1A and S1). Images of intact lymph nodes at the time of isolation showed that HEL-specific B cells were distributed together with non-tg B cells throughout the follicles (0 min in Figures 1A, 1B, and S1). Within 1 h of imaging, accumulation of HEL-specific B cells along the rim of the follicles became evident, whereas non-tg B cells remained uniformly distributed in the follicles (Figure 1B and 1C; Videos S1 and S2). Analysis of individual naive non-tg B cells revealed that these cells moved in an apparently random manner in the follicle with a velocity of approximately 7 μm/min (Figure 1D and 1E), in agreement with previous studies [11]. By contrast, HEL-binding B cells tracked 1–3 h after antigen injection showed reduced velocities, moving at about 4 μm/min (Figure 1E). Within 6 h after antigen challenge, the velocities of activated B cells were restored, and by 24 h, the cells became more motile than naive B cells (Figure 1F; Video S3).

To determine whether antigen-engaged B cells accumulated at the B/T boundary because of directional movement or as a result of random migration followed by retention, we tracked the migration of HEL-specific and non-tg B cells. Of 44 HEL-specific cells that could be tracked for more than 30 min, 22 cells migrated to reach the B/T boundary (Figure 1C, tracking data for 12 HEL-specific cells are shown, with seven of the cells reaching the boundary), whereas of 20 non-HEL-specific cells, only four were found at the boundary after 30 min (Figure 1D, tracking data for 11 non-tg cells are shown, with two cells reaching the boundary). Of the Ig-tg B cells that failed to localize to the boundary, 11 cells were only marginally displaced from their starting positions (maximum displacement <20 μm; two examples are shown in Figure 1C as green and light-purple tracking lines). The remaining cells (11/44) did show substantial migration despite failing to move to the boundary (maximum displacement >20 μm; examples shown in beige, dark-brown, and yellow tracking lines in Figure 1C). To quantify the directionality of migration, we constructed double-logarithmic plots of the net displacement of cells from their starting point against their cumulative path length. In the case of linear motion, a slope of one is expected, whereas the net displacement increases as the square root of path length for random motion, resulting in a slope of 0.5 on log/log axes. The HEL-engaged cells that reached the boundary showed a slope closer to one than either HEL-engaged cells that failed to move to the boundary or naive cells (Figure 1G). These data indicate that the migration path taken by antigen-engaged B cells that reached the boundary was closer to a straight line than that of cells not reaching the boundary or of naive B cells, suggesting that migration to the boundary was directional.

**Figure 2:**
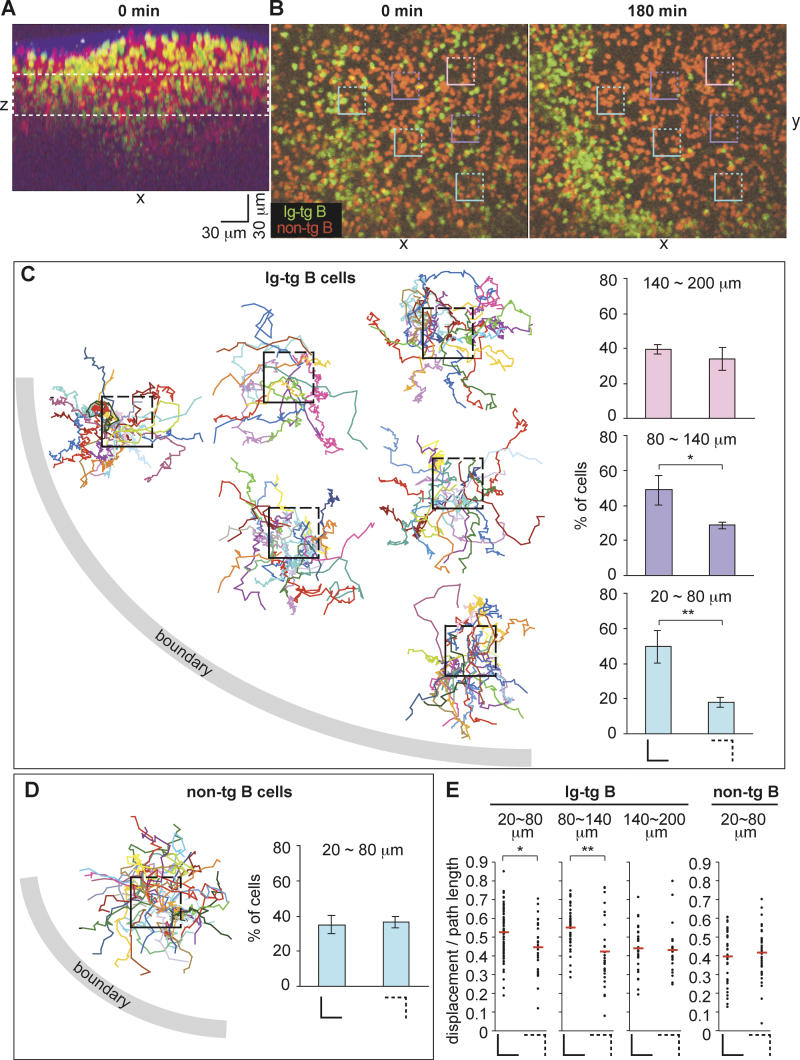
Relationship between Directionality of Ag-Engaged B Cell Migration and Distance from the Follicular Boundary (A) The *xz* projection view of an image stack collected immediately prior to time-lapse imaging, demonstrating the location of the B cell follicle containing transferred B cells (green and red). The collagen-rich lymph node capsule is visualized by second harmonic emission (blue). The dashed white rectangle shows the region used in the time-lapse image analysis. (B) Time-lapse images of HEL-engaged Ig-tg B cells (green) clustering at the follicle–T zone boundary and naive non-tg B cells (red) in the follicle. The 0-min image is approximately 1 h after antigen exposure (see Video S2). Square boxes indicate regions used for directionality analysis, shown in (C). Scale bar is as shown in (A). (C) Tracks of antigen-engaged B cells originating from 30-μm follicular cubes. The centers of the boxes are placed proportionally to the actual positions of the boxes shown in (A), with each box corresponding to a 30 μm × 30 μm × 30 μm cube. Tracks cover 8 to 95 min. The histograms show the percentage of cells that moved across the sides of the square (solid lines) that face the boundary (left histograms) and the percentage of cells that moved across the opposite sides (dashed lines) of the squares (right histograms). The bottom, middle, and top histograms show the results with cubes that are 20–80, 80–140, and 140–200 μm away, respectively, from the boundary. Data shown in the histograms are pooled from three experiments. *, *p* < 0.05; **, *p* < 0.01. (D) Tracks of naive B cells originating from inside of the middle blue box shown in (A). Tracks cover 5 to 25 min. The histogram shows the percentage of cells that moved across the solid or dashed sides of cubes that are 20–80 μm away from the boundary. Data shown in the histogram are from three experiments. (E) The dot plots show ratios of the displacement to the path length of 8-min tracks of antigen-engaged B cells (left three graphs) or naive B cells (far right graph) originating from the cubes described in (B–D). The distances from the cubes to the boundary are indicated above the graphs. The left and right plots in each graph are the data of tracks that cross the solid and dashed sides of the cubes, respectively. All (100%) of Ig-tg B cells and 94% of non-tg B cells that crossed the sides of the cubes could be tracked for 8 min. The means of each data group are shown as red bars. *, *p* < 0.05; **, *p* < 0.01.

A difficulty with measuring migration of activated B cells to the B/T boundary was that the cells being tracked would frequently leave the imaging volume before reaching the boundary. To further quantify directionality and also test its relationship to distance from the boundary, cells that migrated through randomly selected cubic volumes of follicle were tracked for short periods to determine whether they were migrating toward or away from the B/T boundary (Figure 2). We defined 30 μm (*x*-axis) × 30 μm (*y*-axis) × 30 μm (*z*-axis) cubes at various positions within follicles that had been imaged from 1 to 3 h after antigen injection, and the migration of cells emerging from each cube was tracked. The *xy* positions of the follicle–T zone boundary were not greatly changed throughout the 50-μm *z* depth (not shown), and we therefore plotted the cell tracking lines in the *xy* coordinate of the *z*-projection view and counted the number of cells that crossed the sides of the square facing the follicle–T zone boundary (Figure 2B and 2C, sides shown as solid lines) versus those that crossed the opposite sides (Figure 2B and 2C, sides shown as dashed lines). For boxes located 20 to approximately 80 μm away from the B/T boundary (shown in blue in Figure 2B), 50% of the cells emerged across the sides facing the boundary whereas 18% migrated across the opposite sides (Figure 2C). Similarly, for the boxes located 80 to approximately 140 μm away from the follicular boundary (shown in purple in Figure 2B), more cells migrated across the two sides facing the boundary than across the other sides (Figure 2C). However, for boxes located 140 to approximately 200 μm away from the boundary (pink box in Figure 2B), the number of cells that migrated across the sides facing the boundary was not significantly higher than the number of cells that migrated across the opposite sides (Figure 2C). In contrast, the fraction of non-tg B cells that migrated across the sides of the box facing the B/T boundary versus the other sides was essentially even for every box examined, showing no directionality of migration toward the boundary (Figure 2D). Although the B/T boundary was also located below the box, the number of cells leaving via the top and bottom of each box was not included in this analysis because the *z* distance to the boundary could not be accurately determined in two of the experiments. However, as with the above observations, we observed a tendency for more cells to leave via the bottom of the boxes than via the top (data not shown). Plots of displacement over path length for antigen-engaged B cells emerging from each of the cubes revealed that when cells were within approximately 140 μm of the boundary, they showed less turning when migrating toward the boundary than when migrating away from the boundary (Figure 2E). By contrast, non-antigen-engaged B cells and antigen-engaged B cells that were more distant from the boundary showed low displacement to path-length ratios irrespective of their direction of migration (Figure 2E). Taken together, these observations indicate that when antigen-engaged B cells are within approximately 140 μm of the B/T boundary, they are able to conduct directional migration toward the boundary.

### Detection of a CCL21 Gradient

**Figure 3:**
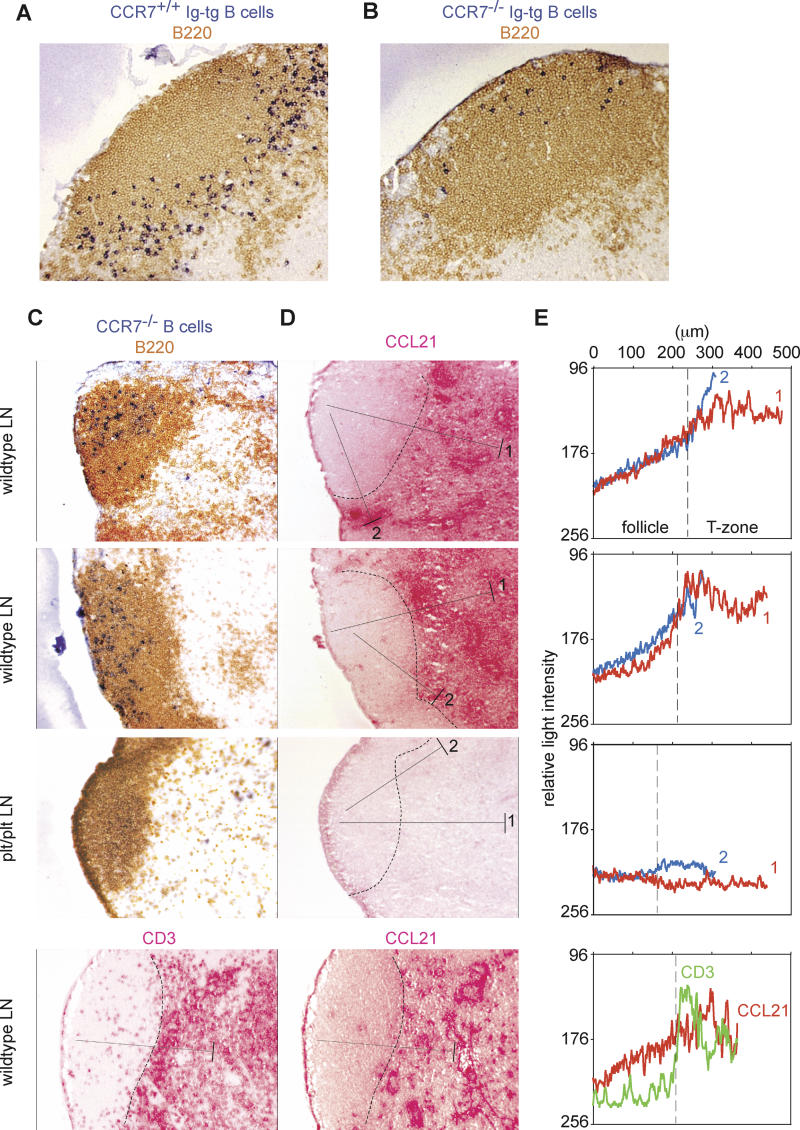
CCL21 Concentration Gradients in B Cell Follicles in Lymph Nodes (A and B) Lymph node sections of wild-type mice that received CCR7^+/+^ Ig-tg (A) or CCR7^−/−^ Ig-tg B cells (B) and 1 mg of HEL intravenously for 6 h, stained to detect all B cells (brown) and HEL-binding B cells (blue). (C) B cell (upper three panels) or T cell (lower panel) staining of lymph node sections from wild-type and *plt/plt* mice as indicated. The wild-type lymph nodes shown in the upper panels were from Igh^a^ mice that had received CCR7^−/−^ Igh^b^ B cells 1 d before. Staining was to detect all B cells (B220, brown) and transferred CCR7^−/−^ B cells of the Igh^b^ allotype (dark blue) or T cells (CD3, red) as indicated. (D and E) Detection of CCL21 concentration gradients in follicle. In (D), adjacent sections were stained for CCL21. The dashed lines indicate the B/T boundary determined by the distribution of B220 staining. The pixel intensity at each position along the filled line extending from the follicle into the deep T zone (line 1) or the interfollicular T zone (line 2) was averaged across 50 μm perpendicular to the line (the width shown by the bar at the T zone end of each line) and plotted against distance from the follicular end of the line (E) in red for line 1 and blue for line 2. As a negative control, the pixel intensity was determined along a line located in the equivalent location in a CD3-stained section (bottom panel of [C] and shown as a green line in [E]). Pixel intensity was measured using Metamorph software after converting colored pixels to gray scale. A decrease in transmitted light intensity indicates an increase in CCL21. Data are representative of more than five lymph nodes from two mice of each type.

As observed previously for cells in the spleen [6], CCR7 was required for antigen-engaged B cells in lymph nodes to localize at the B/T boundary, shown by comparing the distribution of Ig-tg B cells from CCR7^−/−^ and CCR7^+/+^ mice in HEL-injected recipients (Figure 3A and 3B). We also observed that in the absence of antigen-receptor engagement, CCR7^−/−^ B cells tended to localize in regions of lymph node follicles that are distal to the T zone (Figure 3C), suggesting that CCR7 is required for naive B cells to migrate efficiently through the region of the follicle near the T-zone border. The CCR7 ligand CCL21 is abundantly expressed within the T zone [23]. To test whether a CCL21 gradient could be detected extending from the T zone into the follicle, we performed immunohistochemical staining of adjacent lymph node sections for a B cell marker (B220), to locate the boundary between the follicle and the T zone (Figure 3C), and for CCL21 (Figure 3D). CCL21 was concentrated in the T zone as expected, but staining was also observed in regions of B cell follicles that are proximal to the T zone (Figure 3C and 3D). To quantify the CCL21 staining in the follicle, we converted the images to gray scale and measured the intensity of gray pixels along lines drawn from the outer follicle to the T zone (Figure 3E). This approach confirmed that CCL21 was present in an increasing gradient from regions of follicles that are distal to the T zone, to the B/T boundary. An increasing CCL21 gradient could also be observed extending 150–200 μm from inside the follicle to the interfollicular region, the boundary region typically imaged in this study by two-photon microscopy. The gradient was not observed in lymph nodes from *plt/plt* mice, which lack CCL21 expression in lymphoid tissues, although a weak signal was sometimes detected in interfollicular regions, perhaps due to small amounts of CCL21 entering the lymph node via afferent lymphatics (Figure 3D and 3E, “plt/plt LN”) [24]. Quantitation of the staining pattern generated with antibodies against CD3, another protein abundant in the T zone, failed to reveal any evidence of a gradient (Figure 3C–3E, bottom panels), showing that there was minimal diffusion of the fast-red reaction product generated during immunohistochemical staining and confirming that CCL21 was present as a gradient.

### CCR7 Is Required for Directional Migration of Antigen-Engaged B Cells

**Figure 4:**
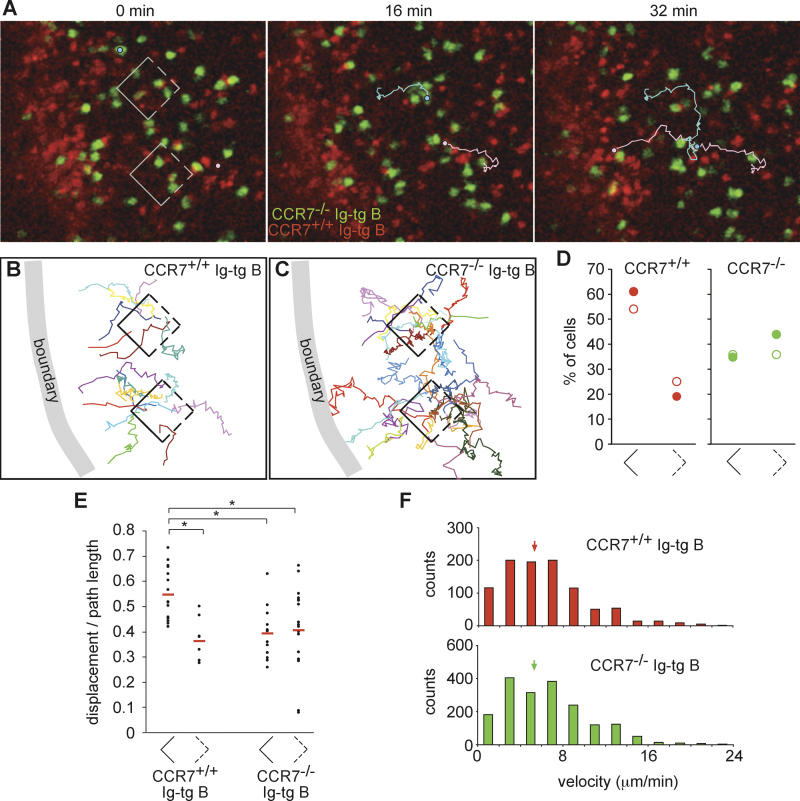
Antigen-Engaged CCR7^−/−^ B Cells Fail to Show Directional Migration toward the Follicle–T Zone Boundary (A) Time-lapse images of HEL-engaged CCR7^−/−^ (green) and CCR7^+/+^ (red) Ig-tg B cells in an inguinal lymph node. The 0-min image is ~3.5 h after antigen exposure (see Video S4). Square boxes indicate regions used for directionality analysis, shown in (B). A CCR7^−/−^ Ig-tg B cell (light blue circle and line) and a CCR7^+/+^ Ig-tg B cell (pink circle and line) are traced as examples. (B and C) Tracks of antigen-engaged CCR7^+/+^ (B) and CCR7^−/−^ (C) Ig-tg B cells originating from 30-μm follicular cubes, analyzed as described in Figure 2. Tracks cover 3–17 min for CCR7^+/+^ cells and 3–22 min for CCR7^−/−^ cells. (D) Dot plot showing the percentage of cells that moved across the solid or dashed sides of the cubes. Filled symbols correspond to the data shown in (A–C) and Video S4, open symbols correspond to data obtained in a second time-lapse movie collected 2.5–3.5 h after antigen injection. The cubes were located approximately 20–80 μm from the site of accumulation of CCR7^+/+^ Ig-tg B cells. (E) The dot plots show ratios of the displacement to the path length of 8-min tracks of antigen-engaged CCR7^+/+^ or CCR7^−/−^ Ig-tg B cells originating from the cubes described in (A–D). The left and right plots for each cell graph are the data of tracks that cross the solid and dashed sides of the cubes, respectively. A total of 74% of wild-type B cells and 81% of CCR7^−/−^ B cells that crossed the sides of the cubes could be tracked for 8 min. The means of each data group are shown as red bars. *, *p* < 0.01. (F) Velocity distribution data for CCR7^−/−^ Ig-tg B cells (green, *n* = 27) and CCR7^+/+^ Ig-tg B cells (red, *n* = 40) tracked 2.5–4.2 h after antigen injection. The data are pooled from two time-lapse movies. Medians are indicated by arrows.

To further test whether directional migration of antigen-engaged B cells to the B/T boundary occurred in response to a CCR7 ligand gradient, we tracked the migration of carboxyfluorescein diacetate succinimidyl ester (CFSE)–labeled CCR7^−/−^ Ig-tg B cells in wild-type recipients 1–4 h after antigen exposure. 5-(and-6)-(((4-chloromethyl)benzoyl)amino)tetramethylrhodamine (CMTMR)–labeled CCR7^+/+^ Ig-tg B cells were co-transferred with the CCR7^−/−^ cells as an internal control. As expected, the CCR7^+/+^ cells were observed to accumulate at the edge of the follicle (Figure 4; Video S4). By contrast, although the CCR7^−/−^ cells moved at a similar velocity as the wild-type cells (Figure 4F), they failed to accumulate at the boundary (Figure 4A; Video S4). Measurements of the number of cells that migrated from the boxed areas shown in Figure 4A–4C revealed that the CCR7^−/−^ cells did not display directional bias in their migration, in contrast to CCR7^+/+^ cells in the same region, which displayed preferential migration toward the boundary (Figure 4B–4D). As well as quantitating the direction in which cells exited from the boxes, we also measured the number of cells entering the boxes. More than twice as many wild-type Ig-tg B cells entered from the sides located away from the boundary as via the sides adjacent to the boundary, whereas approximately equal numbers of CCR7^−/−^ cells entered through the distal and adjacent sides (data not shown), confirming that CCR7^+/+^ but not CCR7^−/−^ cells showed directional migration toward the boundary. As observed above, wild-type B cells that migrated toward the boundary had an increased displacement to path-length ratio compared to cells migrating in the opposite direction (Figure 4E). By contrast, CCR7^−/−^ cells that emerged from the boxes in the direction of the boundary had the same low displacement to path-length ratio as cells that were migrating in the opposite direction. These findings establish that directional migration of antigen-engaged B cells toward the B/T boundary is CCR7 dependent.

### Dynamics of Interactions between Antigen-Engaged B Cells and Helper T Cells

**Figure 5:**
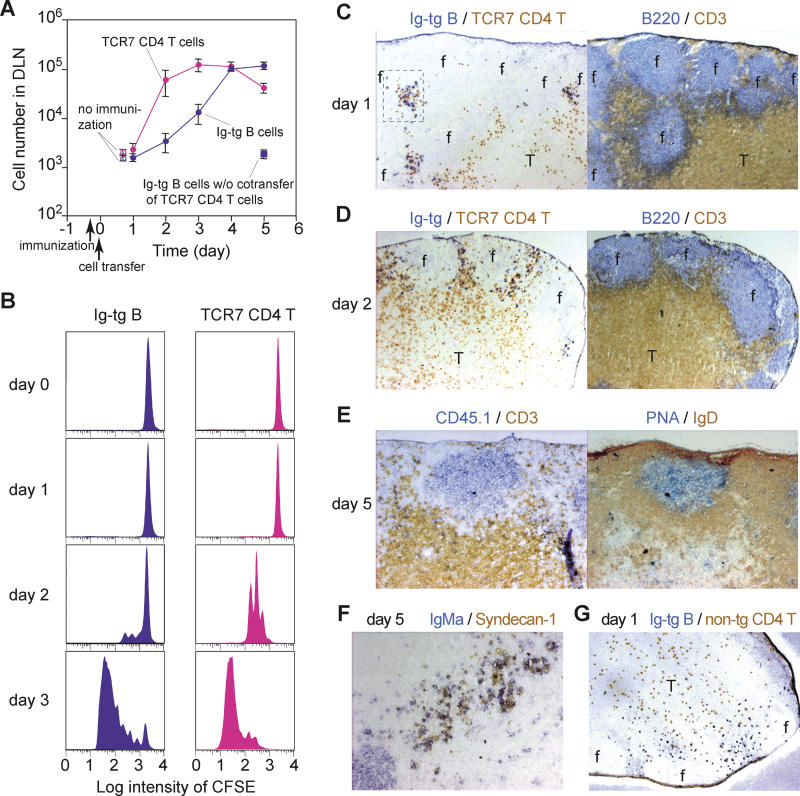
Kinetics of HEL-Specific B Cell Expansion and Differentiation in the Presence of HEL-Specific TCR-Transgenic Helper T Cells (A) Numbers of Ig-tg B cells and TCR7 CD4^+^ T cells in draining (two inguinal) lymph nodes plotted against time after cell transfer. Shown are means ± standard errors of more than three experiments. Immunization of recipient mice with HEL in adjuvant was done 8 h before cell transfer. (B) Time course of B and T cell division in draining lymph nodes, determined by CFSE dilution. (C–F) Immunohistochemical analysis of draining lymph nodes at days 1–5 following immunization, stained as indicated. Note redistribution of B cells into interfollicular clusters in the presence of helper T cells. An enlarged version of the boxed region in (C) is shown in Figure S2. (G) Distribution of Ig-tg B cells at day 1 in the absence of helper T cells, stained as indicated. Objective magnification in (C), (D), and (G), 5×, and in (E) and (F), 10×.

After arrival at the B/T boundary, antigen-engaged B cells interact with activated helper T cells. In order to observe antigen-specific interactions between B cells and helper T cells, we utilized class II (I-A^b^)–restricted HEL-specific TCR-transgenic mice (TCR7; M. Neighbors, S. B. Hartley, and A. O'Garra, unpublished data). The Ig-tg B cells were co-transferred with CD4^+^ TCR7 T cells into recipient mice that had been immunized subcutaneously with HEL plus adjuvant for 8 h (Figure 5). The TCR7 T cells and Ig-tg B cells in draining lymph nodes started proliferating between 1 and 2 d after transfer (Figure 5A and 5B). Consistent with a previous report [7], B cell proliferation was delayed compared to T cell proliferation and peaked 4 to 5 d after transfer. B cell proliferation was not observed if TCR7 T cells were not co-transferred (Figure 5A). By contrast, T cell activation and proliferation occurred even in the absence of transferred B cells (data not shown), consistent with other studies indicating that after injection of antigen in adjuvant, T cells are rapidly activated within the T zone by antigen-bearing DCs [18,25]. One day after transfer, Ig-tg B cells localized in compact clusters with TCR7 T cells in interfollicular regions (Figure 5C). This pattern was distinct from the uniform distribution of Ig-tg B cells along the B/T boundary upon antigen challenge in the absence of transferred T cells (see Figures 3A and 5G) [5], suggesting that B cell clustering is promoted by TCR7 helper T cells. Two days after transfer, when TCR7 T cells had proliferated robustly, Ig-tg B cells remained in interfollicular regions, although the clusters seemed to be less compact (Figure 5D). After 5 d in the presence of antigen and helper T cells, Ig-tg B cells formed germinal centers and also differentiated into plasma cells based on syndecan-1 staining (Figure 5E and 5F).

**Figure 6:**
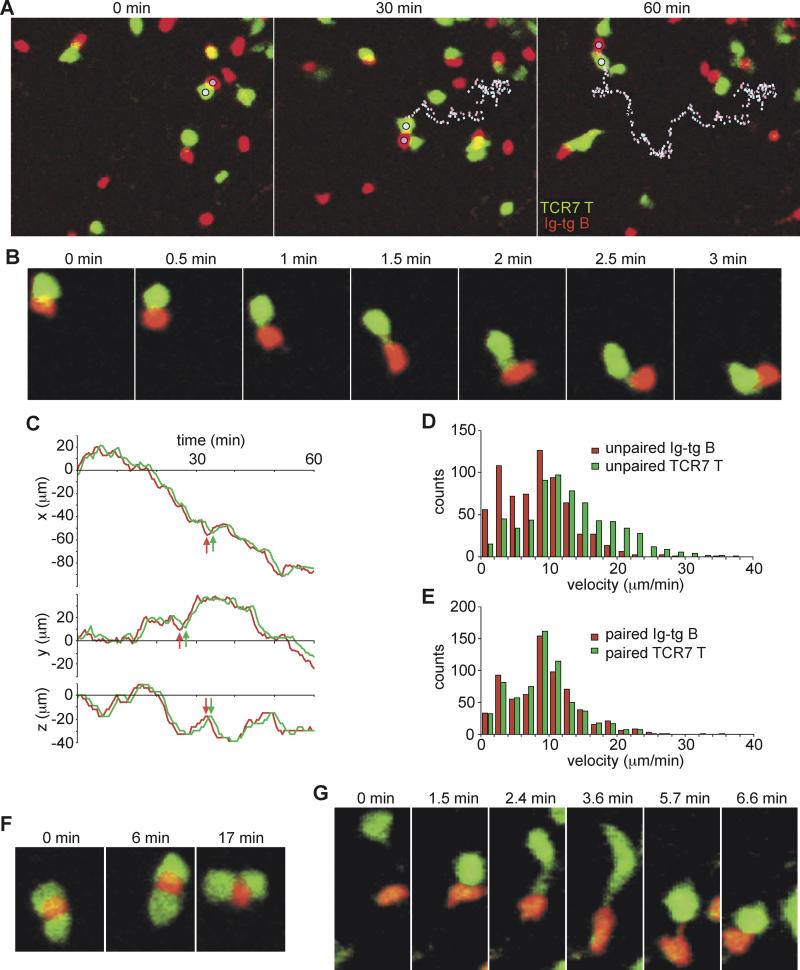
Dynamics of Antigen-Engaged B Cell–Helper T Cell Interactions (A) Time-lapse images of Ig-tg B cells interacting with TCR7 CD4^+^ T cells ~30 h after immunization with HEL in adjuvant, showing T cells moving along behind B cells. The pathways of a B cell (pink dotted line) and a T cell (blue dotted line) remaining bound to each other for more than 1 h are shown (see also Video S5). (B) Time-lapse images showing the dynamics of a B-T conjugate. (C) The *t-x, t-y, t-z* plots of the interacting B (red line) and T (green line) cells traced in (A). Note the B cell makes turns before the T cell (arrows). (D and E) Velocity measurements of unpaired (D) and paired (E) B and T cells, showing that paired T cells slow to the velocity of the B cell. Velocity data are from 16 cells of each type. (F) Time-lapse images of a B cell interacting with two T cells. (G) Time-lapse images showing an encounter of a B cell and a T cell to form a conjugate.

Having established that our adoptive transfer system supported T-dependent B cell antibody responses, we performed two-photon microscopy of fluorescently labeled Ig-tg B cells and TCR7 T cells to analyze the dynamics of B-T interactions in draining lymph nodes. One day after cell transfer, which corresponds to ~30 h after challenge with antigen in adjuvant, Ig-tg B cells and TCR7 T cells were observed in focal clusters and were highly motile. On the basis of immunohistochemical analysis (Figures 5C, 5D, and S2) and the fact that the inguinal lymph nodes were imaged in the same orientation and at similar depths as in Figures 1 and 2, we presume that these clusters were in interfollicular regions. The Ig-tg B cells formed stable conjugates with TCR7 T cells, but typically the conjugate pair remained highly motile (see Videos S5–S7; Ig-tg B cells, red; TCR7 T cells, green). As conjugates moved, B cells always moved in front of helper T cells, and when making turns, B cells turned first and the T cell partner followed (Figure 6A–6C; see also Videos S5 and S6), suggesting that the conjugate pair was led by the B cell partner. Moreover, the instantaneous three-dimensional velocity of B cells in monogamous conjugates (approximately 9 μm/min) was similar to the motility of B cells free of helper T cells (Figure 6D and 6E). In contrast, the motility of T cells in monogamous conjugates (approximately 9 μm/min) was virtually identical to the motility of the activated B cells and lower than the motility of free helper T cells (approximately 14 μm/min; Figure 6D and 6E). Further imaging made at various times between 40 and 64 h after immunization revealed similar dynamics of B-T interaction. Video S7 provides four time-lapse image segments showing the spatiotemporal changes induced in Ig-tg B cell and TCR7 T cell distribution, motility, and interactions from the time of antigen challenge until 64 h after challenge, when extensive T cell division is occurring.

Although stable B-T conjugates were largely monogamous, polygamous conjugates consisting of one B cell sandwiched by multiple T cells were also observed. The polygamous conjugates were less motile than the monogamous conjugates (Figure 6F; Video S8). On the other hand, we never observed a single T cell forming stable conjugates with multiple B cells for more than 5 min. In instances when one T cell contacted multiple B cells, the T cell typically followed one of the B cells within several minutes (Video S9). Some B cells were also observed to switch T cell partners instead of keeping contacts with both T cells (Video S10).

Encounters of B and T cells took place in a variety of ways: T cells approached B cells, B cells approached T cells, and T cells and B cells collided while migrating. During the initial formation of a conjugate pair, T cells sometimes remained attached by a tether to the B cell before rounding up (Figure 6G). As the conjugate pair moved, contact was sometimes maintained by a B cell tether extending from the trailing edge of the B cell to the T cell (Figure 6G; Video S11). Such tethers are consistent with a motile B cell dragging a passive T cell behind it.

**Figure 7:**
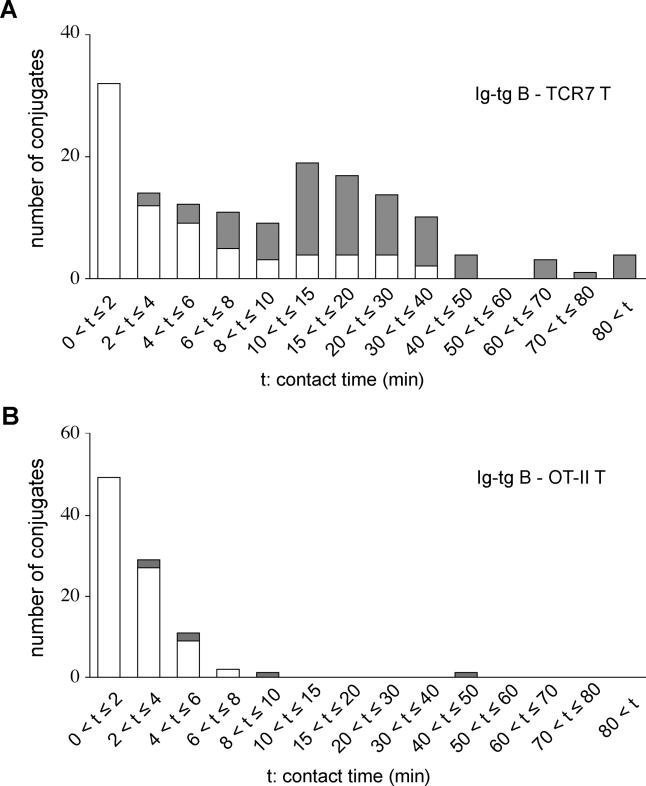
Contact Times of Antigen-Engaged B cell–Helper T Cell Conjugates (A) The histogram shows contact time distribution for Ig-tg B cells and TCR7 CD4^+^ T cells 30 to 50 h after immunization with antigen with adjuvant. Open bars show conjugates that were tracked for the duration of contact and shaded bars show conjugates that could not be tracked for the entire period of contact because the cells entered the field as a conjugate, left the field as a conjugate, or both. (B) Contact time distribution for Ig-tg B cells and OT-II CD4^+^ T cells 1 to 2 d after antigen priming. Open and shaded bars as in (A).

To determine whether the formation of stable B-T conjugates was due to cognate-antigen specificity, we examined the interactions that occurred between B and T lymphocytes specific for two different antigens. OT-II CD4^+^ T cells, which have a class II–restricted ovalbumin (OVA)–specific TCR, were transferred together with Ig-tg B cells into recipient mice immunized as for the above studies except using a combination of HEL and OVA. By this approach, both B cells and T cells become activated, but the HEL-specific B cells are not expected to present OVA peptides to the T cells. In contrast to antigen-specific interactions, contacts between Ig-tg B cells and OT-II T cells were short lived (Video S12). Measurements of B-T contact times in time-lapse recordings made 30–50 h after immunization with antigen in adjuvant revealed that many (81/150, or 54%) of the antigen-specific contacts lasted longer than 8 min, whereas the great majority (90/93, or 97%) of the noncognate interactions lasted less than 8 min (Figure 7). Of the 81 stable antigen-specific conjugates that were tracked, at least 15% (12/81) persisted longer than 40 min. It was difficult to determine entire contact periods for many (75/150, or 50%) of the long-lasting antigen-specific conjugate pairs because the cells entered or left the field of view as a conjugate. Of the 75 conjugates in which cell–cell association and dissociation were recorded, 23% (17/75) had a measured contact time of between 8 and 40 min (Figure 7). Some of the conjugates that moved in or out of the field may also fall in this time window, so this measurement is most likely an underestimate. A small number of conjugates remained in the field of view and did not dissociate for the entire 60–90 min imaging period, establishing that B-T interactions can persist for at least 1.5 h (Figure 7).

## Discussion

Using time-lapse two-photon imaging, we have shown that after antigen engagement, B cells reduce their migration speed and display a clear bias in migration toward the B/T boundary in lymph nodes. By contrast, co-transferred CCR7-deficient Ig-tg B cells failed to show preferential migration toward the boundary. Moreover, the CCR7 ligand CCL21 was present as a gradient within the follicle, increasing toward the boundary over a region where B cell migration bias was observed by two-photon imaging. We propose that after antigen capture and CCR7 upregulation, B cells use this long-range chemokine gradient to navigate to the B/T boundary. Within 1 d of antigen encounter, the B cells had increased their motility, and they maintained this behavior while making stable conjugates with helper T cells. Many cognate interactions between antigen-engaged B cells and helper T cells lasted 10 to 40 min, and some interactions persisted for more than 1 h, whereas cells forming noncognate interactions dissociated in less than 10 min. Taken together, these experiments provide evidence of B cell chemotaxis in vivo; they also reveal that interactions between antigen-engaged B cells and cognate T cells are dynamic and may involve multiple, serial contacts.

Chemotaxis has been implicated in many biological processes within multicellular organisms, including positioning of cells during development and homing of leukocytes during immune surveillance and in the immune response. However, despite extensive in vitro evidence in support of directed cell migration along chemoattractant gradients [26], only limited in vivo evidence of chemotaxis has so far been reported [27,28]. Our findings indicate that in lymph nodes, antigen-stimulated B cells migrate up a chemokine gradient toward the B/T boundary, providing an in vivo example of chemotaxis in a multicellular organism.

Our results suggest the following sequence of events during the spatiotemporal reorganization of antigen-engaged B cells within the follicle. During the first few hours after encountering antigen, B cells upregulate CCR7 [6]. Initially, the random motility of B cells promotes intermingling of individual cells within the follicle and allows them to get close to the follicle edge. Once B cells are within 80 to approximately 140 μm of the edge, antigen-engaged B cells begin to respond via CCR7 to the CCL21 gradient and move in a directed manner toward the B/T boundary. Deviations from a linear migration path occur due to collisions with randomly migrating naive B cells and small-scale irregularities in the CCR7 ligand gradient. At the boundary region, the cells continue to migrate extensively. When activated CD4 T cells are present, the B cells and T cells co-cluster in interfollicular regions, areas that also contain many DCs. Antigen-engaged B cells form conjugate pairs with T cells and continue to migrate actively in the interfollicular region, pulling the T cell partner behind. Activated B cells remain within these regions for 1–2 d, most likely undergoing many consecutive 10–40 min interactions with helper T cells before B cell proliferation begins.

Despite the large displacement of many of the antigen-engaged B cells, all of the cells showed reduced migration speeds in the first hours after antigen-injection, a behavior that may be due to enhanced adhesion of B cells to follicular stromal cells or other neighboring cells. B cell–receptor (BCR) stimulation increases the avidity of integrins α_L_β_2_ and α_4_β_1_, promoting increased attachment of B cells on intercellular adhesion molecule-1 (ICAM-1) and vascular cell adhesion molecule-1 (VCAM-1) [29,30], integrin ligands that are expressed by follicular dendritic cells and neighboring B cells [31,32]. Early after antigen challenge, some HEL-engaged B cells in the part of the follicle distal to the T-zone were not significantly displaced during 30-min imaging experiments. In this regard, it is notable that antigen-receptor stimulation in T cells induces transient α_L_β_2_ activation that persists for about 30 min [33]. In vitro studies have indicated that as integrin-mediated adhesiveness increases above a critical threshold, cell motility decreases [34]. The BCR signaling may also affect intrinsic motility of B cells by rearranging their cytoskeleton through the Vav–Rho–GTPase pathway [35].

The finding that many antigen-engaged B cells showed CCR7-dependent directional migration toward the B/T boundary region is consistent with the conclusion that the cells are migrating in response to the gradient of CCL21 that extends from the T zone into the follicle. The factors determining the distribution of CCL21 are not clear, but it seems likely that the chemokine is distributed along stromal cells that are present throughout the follicle [36]. Previous studies have shown that T-zone CCL21 is concentrated on the stromal cell surface [23,37]. We also consider it likely that the naive B cells migrating through the part of the follicle that is proximal to the T zone bind and transiently display CCL21 in a manner that permits engagement of receptors on other B cells. In addition to displaying directional migration in a CCR7-dependent manner, some antigen-engaged B cells appear to arrive at the B/T boundary by random migration from the follicle and, possibly, other locations because some CCR7-deficient cells were found at the boundary. However, CCR7-deficient cells fail to accumulate at the boundary over time [6], and it is likely that CCR7 functions both to promote directional migration of antigen-engaged cells and to help retain cells in the boundary region. A dense network of stromal cells is present on the T-zone side of the boundary, an area known as the outer cortex [38] or cortical ridge [37], and it will be important to determine whether these cells contribute to retaining antigen-engaged B cells in this region. The finding that antigen-engaged B cells at the B/T boundary are highly motile indicates that the mechanisms retaining cells in this region are unlikely to be solely adhesion based. At the start of our imaging experiments, the transferred B cells had equilibrated in the host for at least 1 d and the majority of Ig-tg B cells were located within lymphoid follicles. Because soluble HEL rapidly gains access to lymphoid follicles [39], we assume that most of the B cells in our studies are encountering the antigen while migrating within the follicle. Although we believe our findings should be generalizable for B cells encountering any type of follicular antigen, including antigens displayed by follicular dendritic cells, in some cases B cells may encounter antigens in the blood or at the point of entry into the lymph node, and in this situation, movement to the B/T boundary most likely occurs by a different path from the one examined in this study. The B/T boundary region imaged in our experiments corresponds to the boundary of the follicle and interfollicular region. Although our frozen-section analysis suggested that antigen-engaged B cells accumulated similarly along the length of the B/T boundary in lymph nodes and spleen, it remains possible that cell behavior will vary in different subregions of the boundary.

Similar to B cells encountering a foreign antigen, autoantigen-binding B cells localize at the B/T boundary [5]. Because their chronic exposure to autoantigen excludes these cells from accessing follicles during their short life span, the term “follicular exclusion” has been used to describe this process for autoreactive B cells. In some cases, autoantigen-binding B cells appear to lodge at the B/T boundary because of reduced expression of CXCR5 rather than increased expression of CCR7 [40]. It will be interesting in future studies to examine the migratory behavior of autoantigen-binding B cells at the B/T boundary.

Unexpectedly, conjugates of antigen-specific B and T cells were found to migrate extensively, being led by B cells. We propose that T cells in the conjugates are not themselves motile but are dragged by B cells through firm adhesion based on the following observations: (1) in B-T conjugate pairs, B cells turn first and T cells follow; (2) trailing T cells in a conjugate are often rounded and yet the conjugate pair is moving; and (3) conjugate pairs move with the velocity of activated B cells. Conjugate velocities (approximately 9 μm/min) were substantially greater than velocities of DC–T cell conjugates (<4 μm/min), although here also the T cells followed along after their antigen-presenting partner [14,18]. In vitro studies have established that T cells become polarized toward their interaction partner, and it seems likely that this reorganization of the cytoskeleton is not compatible with also maintaining motility [41,42]. Our observations are also consistent with the “stop signal” concept that was developed from the finding that T cells stop crawling on artificial lipid bilayers containing MHC class II and ICAM-1 molecules when specific TCR ligands are given [43]. In vitro studies with T cells and a recent study that imaged cell movement in thymic slices by two-photon microscopy showed that motility arrest was caused by TCR-induced elevations in cytosolic Ca^2+^ concentration [44–46]. Contrary to our observations, a recent study using confocal laser scanning microscopy to image interactions between naive DO11.10 T cells and peptide-loaded B cells concluded that B cells were being pushed by actively migrating T cells [47]. However, most of those data were obtained by imaging cells in a collagen matrix, and although this system provides information on the spectrum of B-T interactions that are possible, these conditions differ substantially from the environment within a lymph node, in which much of the collagen is sheathed by stromal cells (reviewed in [36]). Moreover, no BCR stimulus was included in the experiments performed by Gunzer et al. [47], and this could also explain the differences in our observations. By migrating while conjugated with T cells, B cells may continue to survey for helper T cells, a process that could promote T cell exchange. During a response involving a diverse repertoire of T cells, continued surveillance might allow B cells to exchange weakly interacting, low-affinity T cells for cells with higher-affinity TCRs that are likely to provide more robust help [49].

Our results show that many cognate B-T interactions last for more than 8 min, with some interactions lasting more than 1 h, whereas non-antigen-specific interactions between B cells and helper T cells typically last less than 8 min. Interaction for more than 8 min is likely to provide sufficient time for the formation of immature immunological synapses between the interacting B-T interfaces [50,51]. Although we were unable to obtain precise measurements of the persistence times of many of the stable conjugates because of their unexpectedly high motility, we can conclude that at least 21% of the conjugates that last longer than 8 min dissociate within 40 min. Therefore, the development of a stable antigen-specific conjugate does not automatically mean that the conjugate will persist for a period of hours. Reciprocally, at least some of the conjugates persisted for at least 40 min, and some for longer than 80 min. Given that the antigen-receptor specificities on all the B cells and T cells in our system are identical, the basis for the different contact duration is not clear at present. It is also notable that many of the noncognate B-T contacts lasted for several minutes, similar to the duration of noncognate interactions imaged in vitro and an amount of time that may be sufficient for initial clustering and scanning of MHC class II peptide complexes [45,51]. A large fraction of cognate B-T contacts were as short as noncognate contacts, suggesting that many initial antigen-specific contacts fail to achieve adequate signaling and changes in adhesiveness to proceed to stable interactions.

At present little is understood about the cumulative amount of T cell stimulation that is required for ensuring B cell commitment to proliferation and differentiation. Our studies reveal that interactions between B and T cells begin taking place within 1 d of immunization with antigen in adjuvant, whereas T cell–dependent B cell proliferation does not begin for another 12–24 h. Using a slightly different system, Garside et al. reported that cognate B-T contacts were not observed in lymph node sections until 2 d after antigen challenge, but again there was about a 1-d lag before B cell proliferation occurred [7]. Experiments with anti-CD40 antibodies have indicated that prolonged antibody exposure over a period of days is necessary to induce B cell proliferation, although these studies typically did not employ antigen-stimulated B cells [52]. In our studies so far, we have not observed differences in the duration of B-T contacts at day 1 versus day 2 of antigen priming (data not shown). Therefore, at present, we favor the view that most B cells interact consecutively with multiple T cell partners over a period of approximately a day before B cell proliferation is induced.

Instead of being distributed along B/T boundaries, antigen-specific B cells were localized in interfollicular zones when TCR7 antigen–specific helper T cells were present. This clustering was not seen when recipients of Ig-tg B cells alone were immunized with antigen in adjuvant, indicating that clustering was not directly induced by the adjuvant and that the low, endogenous HEL-specific T cell response of the B6 mice was insufficient to cause the relocalization. The relocalization may be caused by the activated TCR7 T cells making chemokines or inducing chemoattractant production by other cells, such as antigen-bearing DCs. Ingulli et al. showed that 18 h after subcutaneous injection of fluorochrome-labeled OVA, a subpopulation of antigen-bearing CD11b^+^ DCs was present in paracortical regions adjacent to follicles [53]. Interfollicular regions are also rich in high endothelial venules, and other studies have indicated that DCs newly arriving in lymph nodes are often found near follicles or clustered around high endothelial venules [14,17,54], and small, soluble antigens can reach these regions via conduits that connect with the subcapsular sinus [25,39]. The congregation of cells in interfollicular regions may also foster B cell–DC interactions, which themselves may be important during T cell–dependent antibody responses [2].

In regions that were not crowded with helper T cells, B cells mainly formed one-to-one contacts with helper T cells. However, antigen-engaged B cells occasionally formed stable contacts simultaneously with multiple helper T cells, especially in areas crowded with helper T cells. These observations suggest that in physiological conditions, antigen-specific B-T interactions are initially monogamous, but polygamous interactions may occur after antigen-specific T cells expand in number. The ability of B cells to form stable interactions with more than one T cell has also been observed in vitro and is consistent with evidence that B cells do not undergo cytoskeleton-dependent polarization toward the T cell [50,51]. This may also be important in allowing the continued motility of the B cell in the B-T conjugate pairs. Future studies are needed to learn whether simultaneous interactions with multiple T cells induce different signaling outcomes in B cells compared to one-to-one interactions. In contrast, we did not find helper T cells forming stable conjugates with multiple B cells simultaneously. Instead, when contact was made with multiple B cells, helper T cells followed only one of the B cells. These findings are in good accord with in vitro studies showing that individual T cells use their cytoskeleton to actively polarize toward a single B cell partner [41,45,50,51]. These observations suggest that each T cell forms one immunological synapse at a time in cognate B-T interactions in lymph nodes.

## Materials and Methods

### 

#### Mice and cells

The HEL-specific Ig-tg mice and cOVA-specific TCR-transgenic mice were of the MD4 line and of the OT-II line, respectively [55,56]. B6.Cg-*Igh^a^ Thy1^a^ Gpi1^a^/J*(B6-Igh^a^) and μMT mice were from the Jackson Laboratory (Bar Harbor, Maine, United States). The HEL-specific TCR-transgenic mice were of the TCR7 line that carries transgenic TCR-αβ-recognizing peptide HEL_74–88_ in I-A^b^ (M. Neighbors, S. B. Hartley, and A. O'Garra, unpublished data). All transgenic lines were crossed with C57BL6 (B6)-CD45.1 mice (Jackson Laboratory). B cells were purified from spleen and lymph node cells of Ig-tg or B6 mice by immunomagnetic depletion of CD43-expressing cells using autoMACS (Miltenyi Biotec, Bergisch Gladbach, Germany). CD4^+^ T cells were enriched from spleen and lymph node cells of B6, TCR7, or OT-II mice by depleting CD8^+^ T cells, B cells, NK cells, DCs, macrophages, granulocytes, and erythrocytes (Miltenyi Biotec). Purity of B and T cells from B6 mice and transgenic BCR-positive B cells from Ig-tg mice was more than 90%. Purity of Vβ3^+^CD4^+^ T cells from TCR7 mice and Vα2^+^Vβ5^+^CD4^+^ T cells from OT-II mice was 70% to 80% because of the presence of CD4^−^CD8^−^ T cells in the periphery. After adoptive transfer, however, transgenic TCR-positive CD4^+^ T cells were more than 90% of transferred cells in recipient lymph nodes. CCR7^−/−^ mice [57] were tenth-generation backcrossed to B6 mice. CCR7^−/−^ Ig-tg B6 mice were frequently found to become stunted and sickly by 4–6 wk of age and could not be used as B cell donors. To generate sufficient B cells for transfer experiments, irradiated B6 or B cell–deficient (μMT) mice were reconstituted for 6–10 wk with 80% CCR7^−/−^ Ig-tg bone marrow and, to provide a source of wild-type T cells, 20% μMT bone marrow. Bone marrow chimeras were made as described by Reif et al. [6].

#### Flow cytometric analysis

Analyses of purified cells and transferred cells were performed on a FACSCalibur (Becton Dickinson, Palo Alto, California, United States). All antibodies were purchased from BD Pharmingen (San Diego, California, United States). For proliferation analysis of antigen-specific cells in lymph nodes, 6–10 million purified Ig-tg B cells and 5–8 million purified TCR7 CD4^+^ T cells were labeled with 7 μM CFSE (Molecular Probes, Eugene, Oregon, United States) for 25 min at 37 °C and transferred intravenously into B6 mice that had been immunized subcutaneously in the flank and the base of the tail with 200 μl of 1.3% alum emulsion containing 400 μg of HEL and 4 μg of recombinant murine TNFα (R&D Systems, Minneapolis, Minnesota, United States) 6–8 h before cell transfer. At 20–24 h after cell transfer, the animals were given a second intraperitoneal injection of 1 mg of HEL in saline. The superficial inguinal lymph nodes were removed after sacrificing the recipient mice and chopped in medium containing 1.6 mg/ml bovine collagenase type IV (Worthington Biochemical, Lakewood, New Jersey, United States) and 50 μg/ml bovine DNase I (Sigma, St. Louis, Missouri, United States). After 30 min incubation at 37 °C, the cell suspension was mashed through 70-μm filters and immunofluorescently stained to identify CD45.1^+^ populations of transferred cells.

#### Immunohistochemistry

Cryostat sections (7–8 μm) of lymph nodes were fixed and stained as previously described [58]. CCL21 staining was performed with goat anti-mouse CCL21 antibody (R&D Systems) and biotinylated donkey anti-goat IgG antibody (Jackson ImmunoResearch, West Grove, Pennsylvania, United States). Biotin was detected with an ABC-AP kit (Vector Laboratories, Burlingame, California, United States). CCR7^−/−^ B cells transferred into B6-Igh^a^ mice were detected by biotinylated anti-IgM^b^ and anti-IgD^b^ antibodies (BD Pharmingen). PNA was from Sigma; anti-IgD was from The Binding Site (Birmingham, United Kingdom); and syndecan-1 was from BD Pharmingen. Ig-tg B cells were detected by HEL binding as previously described [6] or by staining for CD45.1 or IgM^a^. CFSE-labeled CD4^+^ T cells were stained with anti-fluorescein HRP antibody (PerkinElmer, Boston, Massachusetts, United States). All other antibodies were from BD Pharmingen.

#### Two-photon imaging and analysis

For imaging antigen-engaged B cells relocalizing in the B/T boundary, 20–30 million wild-type Ig-tg B cells and 5–10 million B6 B cells were labeled with 10 μM CFSE and 10 μM CMTMR (Molecular Probes), respectively, and transferred to B6 mice. In two experiments, Ig-tg B cells were labeled with CMTMR, and this gave similar results (not shown). For experiments with CCR7^−/−^ Ig-tg B cells, 30–70 million cells were purified from approximately ten bone marrow chimeric donors, labeled with CFSE, and injected into single recipients that also received wild-type CMTMR-labeled Ig-tg B cells as above. Flow cytometric analysis of recipient lymph node cells established that more than 90% of the CFSE^+^ cells were HEL-binding CCR7^−/−^ Ig-tg B cells. One to 2 d after cell transfer, recipient mice were intravenously injected with 1.5 mg of HEL in saline. One, 5, or 18 h after HEL injection, superficial inguinal lymph nodes were isolated, maintained in 36 °C medium bubbled with 95% O_2_/5% CO_2_, and imaged through the capsule in a region distal to the efferent lymphatic by multi-dimensional (*x, y, z,* time, and emission wavelength) two-photon microscopy [11]. For time-lapse image acquisition, some experiments were performed exactly as described by Miller et al. [11]. In other experiments, each *xy*-plane spanned 240 μm by 288 μm at a resolution of 0.6 μm per pixel, and images of 18 *x*y-planes with 3-μm *z* spacing were formed by averaging ten video frames, using emission wavelengths of 500–540 nm (for CFSE-labeled cells), 567–640 nm (for CMTMR-labeled cells), and 360–440 nm (to detect second harmonic emission) every 30 s. For orientation purposes, *z* stacks of up to 200 μm were collected. For imaging B-T interactions, 6–10 million Ig-tg B cells and 5–10 million TCR7 or OT-II CD4^+^ T cells were labeled with 10 μM CSFE and 10 μM CMTMR, respectively, and transferred to B6 mice that had been immunized 8 h before transfer. In two experiments, the dyes were switched, and this gave similar results (not shown). Immunization was as described above for cell proliferation analysis. One day or in some cases 2 d after cell transfer, superficial inguinal lymph nodes were imaged as described above. Image acquisition was performed by Metamorph (Universal Imaging, Marlow, United Kingdom) or Video Savant software (IO Industries, London, Ontario, Canada). Three-dimensional cell tracking was performed using Metamorph software with manual tracking of individual cells between each 3-μm *z*-plane. In any instance where two cells in a given set of adjacent *z*-planes were indistinguishably overlapping, we excluded them from the analysis.

## Supporting Information

Figure S1 Projection Views of Image Stacks Collected before and after Time-Lapse Image Analysis The *xz, yz,* and *xy* projection views of image stacks collected immediately prior to time-lapse imaging (A), or immediately after imaging (B), demonstrating the location of the B cell follicle containing transferred Ig-tg (green) and non-tg (red) B cells. The collagen-rich lymph node capsule is visualized by second harmonic emission (blue). The lymph node was isolated for imaging 1 h after HEL injection and imaging was performed for 3 h. The relocation of the antigen-engaged (green) B cells to the rim of the follicle can be seen most clearly in the *xz* projection view in (B). The time-lapse movie collected from this follicle corresponds to the third dataset included in Figures 1 and 2. Objective magnification, 20×.
(6.4 MB TIF).

Figure S2 Enlarged View of an Interfollicular Cluster of Ig-tg B Cells and TCR7 T Cells in the Draining Lymph Node 1 d after Immunization Ig-tg B cells are shown in blue and TCR7 T cells are shown in brown. The area shown corresponds to the boxed region in Figure 5C. Arrows indicate examples of interactions between antigen-specific B cells and T cells.
(4.6 MB TIF).

Video S1 Relocation of Antigen-Engaged B Cells from the Follicle to the B/T Boundary Time-lapse image sequence shows Ig-tg B cells (green) and non-tg B cells (red) in a lymph node approximately 1–3 h after HEL-antigen challenge. The white circle highlights an Ig-tg B cell that moves to the B/T boundary. Time indicated as h:min:s. Each image is 210 × 180 μm and projects 51-μm *z* stacks. Time compression is 300×.
(3.1 MB AVI).

Video S2 Second Example of Antigen-Engaged B Cell Relocalization from the Follicle to the B/T Boundary Time-lapse image sequence shows Ig-tg B cells (green) and non-tg B cells (red) in a lymph node approximately 1–4 h after HEL-antigen challenge. Time indicated as h:min:s. Each image is 288 × 240 μm and projects 51-μm *z* stacks. Time compression is 300×.
(8 MB AVI).

Video S3 Motility of Antigen-Engaged B Cells Localized on the B/T Boundary Time-lapse image sequence shows Ig-tg B cells (green) and non-tg B cells (red) 18–19 h after antigen challenge. Time indicated as h:min:s. Each image is 210 × 180 μm and projects 51-μm *z* stacks. Time compression is 300×.
(1.6 MB AVI).

Video S4 Migration of Antigen-Engaged Wild-Type but Not CCR7^−/−^ B Cells to the B/T Boundary Time-lapse image sequence is shown as a montage to facilitate improved tracking of the two B cell types, with CCR7^−/−^ Ig-tg B cells shown in the upper panel, wild-type Ig-tg B cells in the center panel, and the overlay of both cell types in the lower panel (CCR7^−/−^, green; wild-type, red). Image sequence corresponds to approximately 3.5–4.2 h after HEL-antigen challenge. Time indicated as h:min:s. Each image is 180 × 150 μm and projects 51-μm *z* stacks. Time compression is 300×.
(2.5 MB AVI).

Video S5 Interactions of HEL-Specific B Cells and HEL-Specific Helper T Cells within the Lymph Node Time-lapse image sequence shows Ig-tg B cells (red) and TCR7 transgenic T cells (green) ~30 h after challenge with antigen in adjuvant. Time indicated as h:min:s. Each image is 200 × 163 μm and projects 51-μm *z* stacks. Time compression is 270×.
(3.9 MB AVI).

Video S6 Migration Dynamics of B-T Conjugates within the Lymph Node Time-lapse image sequence shows Ig-tg B cells (red) and TCR7 transgenic T cells (green) ~30 h after challenge with antigen in adjuvant. Time indicated as h:min:s. Each image is 135 × 114 μm and projects 51-μm *z* stacks . Time compression is 300×.
(1.9 MB AVI).

Video S7 Spatiotemporal Changes Induced in Ig-tg B Cell and TCR7 Transgenic T cell Distribution, Motility, and Interactions after Antigen Challenge The video contains four time-lapse image segments representing initial random motility and segregation in the absence of cognate antigen (segment I), and three times after challenge with HEL in adjuvant: ~40 h (segment II), ~52 h (segment III), and ~64 h (segment IV). B cells shown in red; T cells shown in green. The yellow box in segment IV highlights a representative T cell blast that migrated into the imaging volume, paused, rounded up, and divided. As the daughter cells regain motility and move apart, long membrane tethers can be observed trailing behind. Scale bar = 50 μm; time compression is 450×.
(4.5 MB AVI).

Video S8 Time-Lapse Image Sequence Showing Migration of One HEL-Specific B Cell Simultaneously Interacting with Multiple HEL-Specific Helper T Cells B cells shown in red; T cells shown in green. Time indicated as h:min:s. Each image is 60 × 50 μm and projects 30-μm *z* stacks. Time compression is 300×.
(2 MB AVI).

Video S9 Time-Lapse Image Sequence Showing Brief, Simultaneous Interactions of One HEL-Specific Helper T Cell with Two HEL-Specific B Cells B cells shown in red; T cell shown in green. In this particular movie, it appears that the T cell switches partner before and after the multipartite interaction. Time indicated as h:min:s. Each image is 40 × 45 μm and projects 51-μm *z* stacks. Time compression is 270×.
(547 KB AVI).

Video S10 Time-Lapse Image Sequence Showing a HEL-Specific B Cell Switching HEL-Specific Helper T Cell Partners B cells shown in red; T cells shown in green. Time indicated as h:min:s. Each image is 40 × 35 μm and projects 51-μm *z* stacks. Time compression is 270×.
(979 KB AVI).

Video S11 Encounters between HEL-Specific B and T Cells after Antigen Challenge Time-lapse image sequence sequentially showing three examples of HEL-specific B cells (red) encountering HEL-specific helper T cells (green) to form conjugates. Arrows are included to highlight the initial tethering points between cells. Time indicated as h:min:s. Each image is 50 × 50 μm and projects 24-μm *z* stacks. Time compression is 270×.
(2.4 MB AVI).

Video S12 Migration Dynamics of HEL-Specific B Cells and OVA-Specific T Cells within the Lymph Node Time-lapse image sequence showing the brief interactions of HEL-specific B cells (red) and OVA-specific helper T cells (green) ~30 h after combined immunization with HEL and OVA in adjuvant. Time indicated as h:min:s. Each image is 200 × 163 μm and projects 51-μm *z* stacks. Time compression is 270×.
(1.9 MB AVI).

### Accession Numbers

The LocusLink (http://www.ncbi.nlm.nih.gov/LocusLink/) accession numbers for the gene products discussed in this article are CCL19 (LocusLink 24047), CCL21 (LocusLink 65956), and CCR7 (LocusLink 12775).

## Abbreviations

BCR: B cell receptor
B-T: B cell–T cell
B/T: B-zone–T-zone
CFSE: carboxyfluorescein diacetate succinimidyl ester
CMTMR: 5-(and-6)-(((4-chloromethyl)benzoyl)amino)tetramethylrhodamine
DC: dendritic cell
HEL: hen egg lysozyme
ICAM-1: intercellular adhesion molecule-1
Ig-tg: immunoglobulin-transgenic
non-tg: nontransgenic
OVA: ovalbumin
TCR: T cell receptor
